# Ethical, legal, and social issues (ELSI) and reporting guidelines of AI research in healthcare

**DOI:** 10.1371/journal.pdig.0000607

**Published:** 2024-09-19

**Authors:** Junko Kameyama, Satoshi Kodera, Yusuke Inoue

**Affiliations:** 1 Department of Healthcare Ethics, Kyoto University School of Public Health, Kyoto, Japan; 2 Department of Cardiovascular Medicine, The University of Tokyo Hospital, Tokyo, Japan; 3 The Institute of Medical Science, The University of Tokyo, Tokyo, Japan; Liverpool John Moores University - City Campus: Liverpool John Moores University, UNITED KINGDOM OF GREAT BRITAIN AND NORTHERN IRELAND

This paper aims to (1) summarize the descriptions of ethical, legal, and social issues (ELSI) in major reporting guidelines, identify trends and tendencies, and (2) highlight issues for future researchers and guideline creators.

Medical care is a fundamental aspect of human life, closely linked to their health and well-being. Advances in the research and development (R&D) of medical artificial intelligence (AI) are anticipated to yield numerous benefits. However, concerns regarding incorrect AI design and usage, causing various ethical and human-rights issues for both individuals and society, do arise [1–4]. Policies for research efforts in developing AI have been proposed; however, no consensus on approach selection has been attained [4]. Therefore, this study focuses on “reporting guidelines,” which provide specific result-publication instructions for researchers. The guidelines provide a basis for AI deployment and implementation through the increased transparency and evaluability of processes and data management during R&D [5]. In recent years, AI design and usage have been closely related to ELSI [4]. ELSI is the examination of ethical, legal, and social issues raised by the deployment of new knowledge. This perspective focuses on the impact of new scientific methods and knowledge on the current and future generations. While questioning the role of researchers in ELSI, the position of reporting guidelines requires changes.

Therefore, we review recent reporting guidelines for medical AI research and adopt guidelines with a “checklist” of specific initiatives as the selection criteria. First, six reports registered in the Enhancing the Quality and Transparency of Health Research (EQUATOR) network were selected. The EQUATOR network is an international initiative seeking improvements to the credibility and value of published health-research literature by promoting transparent and accurate reports and disseminating robust reporting guidelines [6]. Furthermore, two other reports from major literature databases (Web of Science and PubMed) were identified and added for a total of eight reporting guidelines [7–14]. While organizing these guidelines from an ELSI perspective, the 11 items of the “Considerations for AI developers,” which append the WHO guidance “Ethics and governance of artificial intelligence for health,” were used [Table 1].

**Table 1:** Ethical, legal, social design, development, and deployment considerations for artificial intelligence (AI) research and health development: Notes for AI developer.

AI design		AI development		AI deployment	
**I**	Clarify the objectives	**VII**	Identify regulatory requirements	**X**	Engage and educate multiple stakeholders for deployment and maintenance
**II**	Engage multiple stakeholders and understand contexts	**VIII**	Establish data management plans	**XI**	Evaluate and improve performance
**III**	Define relevant ethical issues through consultation	**IX**	Adopt standards and best practices		
**IV**	Assess risks				
**V**	Address biases				
**VI**	Privacy by design and privacy by default				

Eleven items are presented as points to keep AI developers in mind. In accordance with the WHO guidelines, the AI research and development process for health is classified into three stages: “AI design,” “AI development,” and “AI deployment,” with relevant ethical considerations at the bottom of each stage.

Ethics and governance of artificial intelligence for health: WHO guidance [4] Annex, Considerations for the ethical design, development and use of artificial intelligence for health “Considerations for AI developers” is prepared by revising

A minimum and maximum of 14 and 58 (average of 25) items were included in the checklists of the 8 reporting guidelines, respectively. "✓" was marked against each reference in the checklist that corresponded to the 11 items proposed by WHO. Additionally, "(✓)" was marked for reports that were not mentioned in the checklist but were mentioned in the text that provided background information and review-process details. For no corresponding descriptions, the checklist was left blank. JK and YI

performed this work, and after both verified the consistency, the results are listed in Table 2. The obtained results, including the clinician’s perspective on SK, were examined, and two major issues were identified.

**Table 2:** ELSI perspectives on reporting guidelines for the R&D of AI technology in healthcare.

Reporting guideline acronym /Date of publication	Targeted readers	Study design	Medical specialization	ELSI*										
				AI design						AI development			AI deployment	
				I	II	III	IV	V	VI	VII	VIII	IX	X	XI
**CLAIM** 2020/05	Authors and reviewers	Diagnostic accuracy studies	Multi-fields	✓				✓	✓	✓	✓			✓
**CONSORT-AI** 2020/09	Investigators, editors, and peer reviewers	Randomised controlled trials (Interventional Study Design)	Multi-fields	✓	✓		✓	✓					✓	✓
**SPIRIT-AI** 2020/09	External reviewers	Clinical Trial Protocol	Multi-fields	✓	✓		✓	✓			✓		✓	✓
**MI-CLAIM** 2020/09	Algorithm designers, repository managers, manuscript writers and readers, editors, and model users	AI modelling	Multi-fields	✓	✓			✓				✓		✓
**CAIR** 2021/05	Clinicians	**—**	Orthopedics	✓	(✓)	✓	✓	(✓)	(✓)	(✓)	✓		✓	✓
**CLEAR Derm** 2021/12	Developers and reviewers	Diagnostic Accuracy	Dermatology	✓	✓	✓		✓	✓		✓	✓		✓
**DECIDE-AI** 2022/05†	Multiple stakeholders‡	Evaluation of human factors in early algorithm deployment	Multi-fields	✓	✓	✓	✓	✓			✓			
**CLEAR** 2023/05	Authors and reviewers	Diagnostic Accuracy	Multi-fields	✓		✓		✓			✓	✓	✓	✓

* From ethics and governance of artificial intelligence for health: WHO guidance [4] Annex, Considerations for the ethical design, development and use of artificial intelligence for health “Considerations for AI developers,” the following 11 items were used in the analysis as ELSI perspectives.

I.Clarify the objectives II.Engage multiple stakeholders and understand contexts III.Define relevant ethical issues through consultation IV.Assess risks V.Address biases VI.Privacy by design and privacy by default VII.Identify regulatory requirements VIII.Establish data management plans IX.Adopt standards and best practices X.Engage and educate multiple stakeholders for deployment and maintenance XI.Evaluate and improve performance

† Three reports are documented over 2021–2023; the 2022 report is the central document that proposes the checklist.

‡ Stakeholders involved in the development of the guidelines. There are no specifications for the targeted reader

## 1. The necessity of efforts for further practical guidance in cooperation with society and patients

Prominently, “V. Address biases” (seven guidelines) were mentioned in the reporting guidelines. However, the purpose and awareness of the issue were unclear in several studies. Recently, the issues of bias surrounding AI have garnered increasing attention in addition to scientific imperatives, owing to its impact on ELSI. For example, biases in data, design and interpretation, and beneficiary exist [4,15]. Considering the diverse concerns surrounding bias, further enhancements are required to support the efforts of researchers.

In addition, as shown in Table 2, an increasingly mentioned item in the guidelines is “III. Defining relevant ethical issues through consultation,” which includes patient and public involvement (PPI) awareness. Precisely, a strong sense of crisis has arisen regarding ELSI, such that the design bias of medical AI and data may directly develop into discrimination and disparities in the recipients of health care [15]. The same purpose is applicable for “II. Engage multiple stakeholders and understand the context.” and “XI. Evaluate and improve performance.” Researchers are required to recognize the growing concerns and incorporate the perspective of potential medical AI targets in their R&D processes. To help researchers smoothly develop such initiatives, highly practical guidance content from guideline creators is needed.

## 2. Limitations of guidelines and the need for researchers to take the initiative

In the AI reporting guidelines for R&D, items with few checks were identified; overall, ELSI items lagged behind while being addressed. Particularly, “VI. Privacy by Design and Privacy by Default” and “VII. Identify regulatory requirements” were significant, and both were met only in one or two guidelines.

Despite the large amount of detailed personal data handled in AI research, reporting guidelines did not include a section addressing data-protection measures. Globally, personal data protection in research activities is highly concerning, and reporting guidelines require the consideration of issues and alerting researchers. However, the legal requirements for personal-information protection may differ from country to country, and reporting guidelines may offer limitations in providing detailed guidance on the matter for individual research reports. While addressing privacy and legal requirements, researchers should not solely rely on the reporting guidelines but should consider proactive consultation with experts and ethical review committees, such that the legal and ethical measures remain relevant to their plans. The point “IX. Adopt standards and best practices” is as mentioned above, and the lack of reference to best practices in R&D was assumed as a limitation of the guidelines for reporting papers.

ELSI surrounding medical AI are evolving and becoming more inclusive, even in the early stages of R&D. The "Reporting Guidelines" not only present the methods to write papers, but also consider the meaning of ELSI guidelines that link researchers, medical care, and patients. A continuous review of reporting guidelines is essential to ensure that medical AI continues to develop with the support of people.

Moreover, as a next step, it is also important to promote harmonization among guidelines, and for reporting guidelines to be linked to discussions on AI practice in the clinical aspect. In doing so, we hope that the ELSI aspect of the discussion, as described in this paper, will be developed in a way that includes the perspectives of many stakeholders.

## Supporting information

S1 File Relevant sections of the guidelines from which the decisions in Table 2 are based. "✓" was marked against each reference in the checklist that corresponded to the 11 items proposed by WHO. Additionally, "(✓)" was marked for reports that were not mentioned in the checklist but were mentioned in the text that provided background information and review-process details. For no corresponding descriptions, the checklist was left blank. JK and YI performed this work.
(PDF)
