# Supplementary material for: Ethical, legal, and social issues (ELSI) and reporting guidelines of AI research in healthcare
Source: PLOS Digit Health. 2024 Sep 19;3(9):e0000607. doi: 10.1371/journal.pdig.0000607 (PMC11412540; doi:10.1371/journal.pdig.0000607)
Supplement: S1 File — "✓" was marked against each reference in the checklist that corresponded to the 11 items proposed by WHO. Additionally, "(✓)" was marked for reports that were not mentioned in the checklist but were mentioned in the text that provided background information and review-process details. For no corresponding descriptions, the checklist was left blank. JK and YI performed this work. (PDF) [file pdig.0000607.s001.pdf]

Supporting document: Relevant sections of the guidelines from which the decisions in Table. 2 are based

Reporting guideline acronym	ELSI										
	AI design						AI development			AI deployment	
	I	II	III	IV	V	VI	VII	VIII	IX	X	XI
**CLAIM**	✓ Item 3				✓ Item 13	✓ Item 12	✓ Item 12	✓ Item 7			✓ Item 39
**CONSORT-AI**	✓ CONSORT-AI 1a/b	✓ CONSORT-AI 2a (i)		✓ CONSORT-AI 19	✓ CONSORT-AI 5 (iii)			✓ CONSORT-AI 25		✓ CONSORT-AI 5 (iv)	✓ CONSORT-AI 5(vi)
**SPIRIT-AI**	✓ SPIRIT-AI 1 (ii)	✓ SPIRIT-AI 6a (i)		✓ SPIRIT-AI 22	✓ SPIRIT-AI 11a (iii)			✓ SPIRIT-AI 29		✓ SPIRIT-AI 11a (iv)	✓ SPIRIT-AI 22
**MI-CLAIM**	✓ Model performance (Part 4):	✓ Part 1: study design			✓ Part 1: study design.				✓ Part 5: model examination.		✓ Reproducibility (Part 6): Tier 2
**CAIR**	✓ TITLE AND ABSTRACT	(✓) No headline (page 514, line 12-17)	✓ DISCUSSION AND OTHER INFORMATION	✓ RESULTS	(✓) Bias and fairness	(✓) Data and privacy	(✓) Methods and results	✓ DISCUSSION AND OTHER INFORMATION		✓ METHODS	✓ METHODS
**CLEAR Derm**	✓ Item 24	✓ Item 19	✓ Item 25		✓ Item 7-9	✓ Item 1-6		✓ Item 1-6	✓ Item 16-19		✓ Item 25
**DECIDE-AI**	✓ Item 1	✓ Item 2	✓ Item 8	✓ Item 6	✓ Item 4b・9a			✓ Item 4a/b			
**CLEAR**	✓ Item 6		✓ Item 8		✓ Item 15・18			✓ Item 53・54	✓ Item 17・18・25	✓ Item 58	✓ Item 51
